# Materia Medica and Therapeutics—Medical Chemistry—Toxicology and Legal Medicine

**Published:** 1861-01

**Authors:** Emil Fischer


### MATERIA MEDICA AND THERAPEUTICS; MEDICAL CHEMISTRY; TOXICOLOGY AND LEGAL MEDICINE.

BY EMIL FISCHER, M.D.

### Materia Medica and Therapeutics.

I.—*Rhus Toxicodendron in Paralysis.* By Dr. Michalowski.

Dr. Michalowski advises the use of fresh rhus toxicodendron in paralysis, and reports the following case in support of his recommendation:—

A farmer, thirty-five years of age, had been suffering for two years with violent rheumatic pain in the bones and joints of the spinal column and of the lower extremities. At last swelling of the joints supervened, and exostoses of the size of a hen's egg appeared at many points of the vertebral column, and two similar tumors, as large as a child's head, on the transverse rami of the pubic bones. The lower extremities of the patient were completely paralyzed; his digestion was bad, and his urine was discharged by drops. After using many different remedies without success, Dr. Michalowski tried the extract of rhus toxicodendron, prepared from the plant, freshly gathered in botanical gardens. He administered one grain of the extract, made into a pill, with half a grain of the leaves, twice daily, and increased the dose to twelve grains of the extract, and ten grains of the leaves. After the patient had taken two ounces of the extract and one ounce of the leaves he was completely cured. During this treatment the swellings of the bones diminished in size; toward the end of the cure the patient had the sensation in his feet and in the sacral region, as if he were pricked by pins; he also had the feeling of a current going from the sacral region down into his feet. Once only, the administration of the medicine had to be interrupted, as the patient complained of pain and a burning sensation in his throat.—(*Allg. Mediz. Centralzeitung*, 1860, No. 48.)

II.—*Cotyledon Umbilicus in Epilepsy.* By Dr. Rodrigues.

This plant, which was described by Dioscorides under the name of *umbilicus veneris*, was employed in the earliest times. Mérat and Delent say that it is diuretic and refrigerant; topically applied it is resolvent. According to Valmont de Bomare it is employed in burns. Salter and Bullar, two English practitioners, were the first to recommend the juice of it in epilepsy, to be given internally in the dose of a teaspoonful twice a day, or the extract in the dose of five grains. The following is an abstract of some cases in which this treatment was employed:—

1. Maria Felicia, of Alpedrinha, forty-six years of age, of nervous temperament, having been much appalled by the assassination of her husband in 1834, was in consequence of it taken, always at the time of full moon, with epileptic attacks of variable intensity and duration, which were not preceded by an *aura* or any other precursory symptom, but were followed for four or five days by vertigo, tinnitus aurium, depression of spirits, and loss of memory. Having entered the hospital in 1852, she had her usual attack during the night. On the next day she was found much changed in her appearance, and having an air of idiocy. Five days afterward she was purged with castor-oil, and immediately put on the use of the juice of cotyledon umbilicus, a teaspoonful every morning and evening. The next attack was missing at the habitual time, and the patient suffered but from vertigo for two days. She continued to take the juice of cotyledon for one hundred and three days without interruption, and had no other epileptic attack ever since.—(*Gazeta Medica do Porto*, 1852.)

2. A young and delicate-looking seminarist, at Santarem, was taken, in April, 1854, with epileptic attacks, which recurred daily at intervals of two to three hours, manifested themselves by convulsions and frightful contortions, and were of variable duration. All remedies having been employed in vain by the physicians of Santarem, and by Dr. Gil Esteves, the latter administered the juice of cotyledon umbilicus, and in two days the patient experienced a decided amelioration.

3. Correia Martins, sub-deacon, thirty-three years of age, of sanguine temperament, had been subject for three years to frequent attacks of epilepsy, which recurred at every new moon. The juice of cotyledon having been taken, the attacks ceased, and had not reappeared a year after the medicine had been resorted to.

4. I. de Matos, fourteen years old, of sanguine-nervous temperament, had been subject for two years to frequent attacks of epilepsy, during which he lost consciousness and struggled in horrible convulsions which rendered him extremely prostrated. The juice of cotyledon having been used for one month, the attacks ceased, and there has been no relapse for five years.

5. A married woman, twenty-six years of age, living in Portalegre, of nervous temperament, who had been nearly drowned when crossing a river in August, 1856, was suddenly taken the next month with difficulty of speaking; she stammered unintelligible words, with contortions and grimaces, her eyes being turned upward; she foamed at her mouth and had convulsions of the extremities; loss of sensation and movement followed for an hour and a half, and, on awaking, loss of memory and extreme fatigue. Other similar attacks supervened, during which she bit her tongue, but which were not announced by any precursory symptom, and had no relation to the phases of the moon. All the antispasmodics prescribed by different physicians were used in vain. The juice of cotyledon having been ordered in December, 1856, the attacks, which then occurred every second day, became soon less intense, and progressively less frequent, recurring at intervals of eleven, fifteen, sixteen, twenty-two, and twenty-nine days; but, the supply of the fresh plant having been exhausted, and the juice and the extract being very liable to change, the patient ceased taking it, and has not been seen since.—(*Gazetta Medica da Lisboa*, 1860, No. 12.)

Although these cases leave much to be desired in regard to precision and details, they permit the conclusion that the fresh juice of cotyledon umbilicus (in preference to the extract) may be employed internally without danger, and that its administration, continued for several months, is useful in some epileptiform affections, and perhaps in hysteria.—(*Archives Générales*, November, 1860.)

III.—*Aquoso-Alcoholic Extract of Olive-leaves in Intermittent Fever.* By M. A. DE ROSEVILLE.

M. de Roseville has used the aquoso-alcoholic extract of olive leaves, according to Aran's recommendation, in two cases of obstinate intermittent fever, and in three cases of facial and frontal neuralgia with very favorable results. He prescribed 0·75 to 1 gramme in the form of pills, divided into three doses, and repeated this dose if the case required it.—(*Gaz. des. Hôp.*, 1860, No. 53.)

IV.—*Sulphide of Antimony in Diseases of the Heart.* By M. FAUCONNET.

M. Fauconnet states that from his eighteenth year he had been subject to affections of the heart, which always followed up some violent exertion, and were then increased and kept up by the moral causes alive at that age. "During the space of fifteen years I have used and abused, without any result, venesection, leeches, baths, digitalis, water, moderate diet, etc. In my twenty-fourth year I had an attack of paludal fever, and the only consequence of its treatment was the addition of gastralgic pains, rendering my existence quite painful. During five-and-twenty years, though removed from the place of infection, I continued to have these pains in the stomach, combined with attacks of the fever, and without abatement of the affection of the heart. In my fiftieth year I commenced to take the sulphide of antimony for these incessant attacks of fever, and before long with the most beneficial result. The first experience I made after the second day of this treatment was the gradual disappearance of the gastralgic pains, which were my greatest dread. The same took place with the fever. Finally, the palpitations and pains of the heart decreased, though slowly, and at last disappeared. After persisting for two months in the use of this medicine, I experienced, for the first time in thirty years, a satisfactory feeling of health."

M. Fauconnet then relates a case of neurosis of the heart, a case of rheumatic endocarditis and hydrocardia, a case of rheumatic neurosis of the heart, with bloody secretions of recent formation, all of which were cured by the sulphide of antimony.—(*Druggists' Circular, from Gazette Médicale de Lyon*, May,1860.)

V.—*Observations on Coca.* By DR. C. HALLER.

With reference to the elaborate treatise on this plant by Mantegazza, (*North Am. Med.-Chir. Review*, March, 1860, p. 340,) as well as to the alkaloid discovered in it by Wöhler, (*North Am. Med.-Chir. Review*, Nov. 1860, p. 1105,) we notice the following of the observations made by the author :—

A feeble infusion of coca-leaves produces a pleasant excitement, followed by sleeplessness; a stronger one keeps off hunger, prevents loss of breath on ascending mountains, dilates the pupil and blunts the sensation of light. The effect from chewing the leaves formed into a ball takes place more gradually. According to Weddell, excessive chewing of coca causes very offensive breath, pale lips, greenish and dull teeth, and an ugly black stain in the corners of the mouth, an unsteady gait, yellow skin, sunken and feeble eyes surrounded by dark-red rings, trembling lips, and a general apathy. Its first injurious effect is enfeebled digestion; then follows a local disease, which is called "opilacion," but of which no description is given; disturbance in the biliary secretion, and especially biliary calculi. Boulimia for animal excrementitious matter is one of the symptoms of the dyscrasia which terminates with dropsy, and finally, death.

The chemical effect of coca-leaves seems to be similar to that of wine and tea, as the natural disintegration of tissues is very much diminished by their use. Coca-leaves resemble hemp in producing dilatation of the pupil; opium, in exalting the strength of the tired system, and in its peculiar effect upon the mind. V. Tschudi recommended them recently again as a nourishing restorative for mariners, as well as for the prevention of the evil effects following the prolonged use of salted provisions.—(*Wien. Zeitschrift*, N. F. iii., 28, 1860.)

### VI.—*Aceton in Catarrhal Affections of the Larynx.* By Dr. H. Itzigsohn.

Dr. Itzigsohn recommends aceton in chronic laryngeal catarrh, affecting persons of middle age, having a slender, but not too long a neck, and a prominent poma Adami, as is frequently observed in tenor singers. The following is given as an appropriate formula for its use: ℞. Acetonis anglici, Spirit. acet. æther. ana ʒij; Aq. Laurocerasi, ʒj; Ol. Millefol., Ol. Salviæ, Ol. Fœniculi æther. ana gtt. v. Ffteen drops to be taken every three hours, upon sugar.—(*Med. Centr. Ztg.* xxix. 38, 1860.)

### VII.—*On the Treatment of Delirium Tremens by Large Doses of Digitalis.* By G. M. Jones, M.R.C.S., Lond. and Edin. *Testimony in favor of this Treatment.* By Drs. Wynn, Williams, Ballard, and Carr.

We copy from the *Medical Times and Gazette* the following interesting communication by Mr. G. M. Jones, Surgeon to the Jersey General Hospital:—

"Having just had an opportunity of showing to some medical friends from London—Mr. Spencer Wells, Dr. Ballard, and Mr. McCrea—the effects of large doses of digitalis in the treatment of a very severe case of delirium tremens, and having been strongly advised by them to make my experience of this mode of treatment known to the profession, I gladly do so by means of the *Medical Times and Gazette.*

"About twelve years ago I was called to see a patient with delirium tremens, residing about a mile from my house, who was almost *in articulo.* I prescribed a dose of chloric ether with tincture of opium; but the wife, who came for the medicine, took, by mistake, a phial containing *one ounce* of tincture of digitalis. I discovered the error, and was horrified when I heard the patient had taken this dose; but no less surprised than pleased when I also heard that, instead of being poisoned, he was very much better. Under ordinary treatment I fully believed he would have died; but after this single dose he rapidly recovered. Profiting by this hint, I began to give digitalis in all the cases of delirium tremens which came under my care in hospital and private practice; and during the last twelve years I have adopted it in at least seventy cases—this effect of drunkenness being very common in Jersey.

"As to the dose, experience has taught me that the best dose is *half an ounce*

of the tincture given in a little water. In some few cases this one dose is enough, but generally a second dose is required four hours after the first. In some cases, but very seldom, a third dose is called for; but this hardly ever need exceed two drachms. The largest quantity I have ever given was *half an ounce* at first, *half an ounce* four hours afterward, and another *half ounce* six hours after that, making an ounce and a half in ten hours.

"As to the effect of these doses, my impression is that the action is on the brain, not on the heart. The pulse, so far from being lowered in force, becomes fuller and stronger, and more regular, soon after the first dose. The cold, clammy perspirations pass off, and the skin becomes warmer. As soon as the remedy produces its full effect, sleep for five, six, or seven hours commonly follows; sleep is the guide as to the repetition of the dose. No action on the kidneys is evinced by any unusual secretion of urine. Sometimes the bowels are slightly acted on, but not commonly. I have never once seen any alarming symptoms follow the use of these large doses of digitalis. The only case I have lost since adopting this treatment, had a tumor in the brain. In three only was other treatment adopted after digitalis had failed to procure sleep; in other words, in sixty-seven out of seventy cases digitalis was the only medicine used, and sixty-six of these patients recovered. I do not mean that these are the exact numbers of those treated; I am certain as to the deaths, but I may have had more recoveries. I am well within bounds in saying seventy cases in twelve years, and that all of them were well-marked cases of delirium tremens. Slight cases of nervous derangement after drinking I have seen in great numbers; but I speak here only of such cases as required active treatment. My previous experience of the results of the treatment by opium, or some of its preparations, by antispasmodics, etc., had certainly been much less successful; the proportion of deaths was larger, and the recovery much less rapid. Again, I have treated more than one patient successfully by digitalis, who, in subsequent attacks elsewhere, has been treated by opium, and died; and in many of the cases in which I have used digitalis successfully, opium had been previously given without any good effect.

"I will only allude to one case in illustration. On September 9, 1860, I was called to see a gentleman, forty-eight years of age, who was in a very alarming state, having been without sleep four days and nights, having been 'muddled' for two months before, and having previously had 'fits of the horrors.' He had been treated by another practitioner by opium in moderate doses, but had become worse, and when I was sent for, it was the opinion of Mr. Spencer Wells and Mr. McCrea, who accompanied me in my first visit, that the case was as bad a one as *they* had ever seen; certainly *I* never saw a worse. The pulse was almost imperceptible; the skin covered with cold, clammy perspiration; the face deadly pale; the lips blue; the hands tremulously grasping the air; the eye expressive of great fear; the mind gone; he was muttering incoherently. With some difficulty I passed half an ounce of tincture of digitalis down his throat, in the presence of my friends. In a few minutes he became more tranquil, the pulse was felt more easily, and we left him. After four hours I found that he had not slept, but he was rather more sensible, less tremulous, and warmer. I accordingly repeated the dose. Three hours after that, as he had been still without sleep, though in other respects improving, I gave two drachms more, making ten drachms in seven hours. After this he had some sleep, and had slept at intervals during the night. The next morning Dr. Ballard saw him, with my other friends, and all of them were much pleased with the great improvement manifested. He was sensible, his fears had disappeared, he was very slightly tremulous; the skin was warm, the tongue moist, and the pulse full and regular at ninety. The heart's sound and impulse were normal; the bowels had acted once, and urine had been passed in natural quantity. After this he took some broth, drank freely of imperial and lemonade, but took no stimuli of any kind, or any other medicine. He slept uninterruptedly for three hours and a half in the afternoon, and at intervals in addition. The next night was a good one; and when he was seen by my friends again the next morning, he was almost well, and calling out for a mutton-chop.

"I trust that this narrative of the results of my experience may induce others to follow what I believe to be a very valuable practical lesson; but I must warn those who do so not to try, as I have done, any smaller doses than those I have recommended. They would not only lose valuable time by so doing, but I believe would do harm. Doses of half a drachm or a drachm do no good whatever; and the pulse, in some cases where I tried them, became intermitting. I have never seen this effect from the larger doses; on the contrary, a feeble intermitting pulse has generally soon become fuller and more regular, proving, I think, as I said before, and as I again wish to impress on the profession, that the curative action is on the nervous system primarily, and not on the organs of circulation."

Since the publication of the above article, Drs. Wynn, Williams, Ballard, and Carr have corroborated the statements of Mr. Jones, in several communications, published in subsequent numbers of the *Medical Times and Gazette*, and have supported their testimony in favor of the treatment of delirium tremens by large doses of tincture of digitalis, by the report of a number of cases. "My own impression," says Dr. Ballard, "from what I have seen and heard from Mr. Jones, is, that in tincture of digitalis we have a true *counter-poison*—an addition to that class of mutually antidotal poisons of which opium and belladonna have been the most recent example. Alcohol is the remedy by which we counteract the depression produced by digitalis; the converse is also true; digitalis is most remarkably an antidote for alcoholism."

Dr. Carr, of Blackheath, who has been acquainted for two years with Mr. Jones's mode of treating delirium tremens, expresses himself in the following manner: "In no case has this treatment failed to produce a relief to all the urgent symptoms—tremors, delusions, and insomnolence. The effect of full doses appears to be directly on the sensorium, acting as a sedative, and at the same time sustaining the failing powers; the wandering eye, the excited and mal-impressed ear, the unduly imaginative brain, the jactitations of the muscular system, approaching in some cases to, and in others passing into, convulsions, all become calm; the creamy tongue cleans; the staggering pulse acquires volume and distinctness; and, indeed, all the evils of mania a potu pass away. In some cases under my care, where the nervous system has been undermined to the extreme by lengthened indulgence, I have combined chloric ether, in doses of thirty minims, giving such every half hour, with three drachms of the tincture of digitalis, repeating the remedies until two ounces of the latter had been taken, when I have ceased for twenty-four hours; after which I have repeated the medicine in less full doses, measuring the quantities needed by the condition of my patient, the amount of sleep obtained, the degree of tranquillity enjoyed, and the state of the pulse, quiet or tremulous." * * * * *
"Lest the somewhat warmly and adversely expressed remarks of Dr. Armstrong, in your impression of last week, on the subject of digitalis, should deter the profession from the use of that remedy in large doses, as recommended by Mr. Jones, of Jersey, I will, with your permission, add another authority to those already adduced. Pereira gave ʒj of the tincture thrice a day for fourteen days without producing any marked effect; and on one occasion two ounces were taken in two doses without giving rise to the slightest inconvenience. No one doubts that digitalis is, alike with many other valuable medicines, a poison; and with an equal conviction that, when administered in proper doses, it is a valuable remedy. It is well known, too, that its effects are cumulative, consequently its exhibition requires to be carefully watched. With this knowledge no prudent man would give doses, toxic beyond a doubt, such as Dr. Armstrong has described were wantonly given to an epileptic. But in delirium tremens, where the nervous system has been undermined by undue stimulation, when the vital forces are at a low ebb, and when the tolerance of the remedy in question has been repeatedly tested by careful observers, medical men may safely administer the comparatively larger doses, not only with impunity, but with the almost certainty of good. Such, at least, has been my experience for the last two years. With your sanction I will, at a future period, further ventilate this subject, giving a few cases in detail, and compare the opium with the digitalis treatment,—the former the more usual, yet the less successful."—(*Med. Times and Gazette*, October, 1860.)

VIII.—1. *On Santonin.* By Professor Falck, of Marburg. 2. *On the same subject.* By Drs. V. Hasselt and Rienderhoff.

Professor Falck communicates in his treatise the result of fifteen experiments made by himself and Dr. Manns, with the view of investigating the physiological effects of santonin. The conclusions at which he arrives are the following: 1. Santonin and santonin-soda are poisons; at the same time it is not to be denied that they are valuable remedies. 2. A solution of santonin in dilute alcohol, introduced in proper quantity directly into the blood, rapidly causes the death of a dog, and undoubtedly also that of any other animal. 3. Injected into the subcutaneous cellular tissue santonin-soda is absorbed into the blood. Also, if introduced into the stomach, santonin as well as santonin-soda may pass into the blood. 4. Under conditions not completely known, santonin as well as santonin-soda are changed, within the animal body, wholly or partly into a substance which is secreted with the urine, and can be demonstrated in the latter by means of caustic alkalies. 5. The change of santonin, respectively santonin-soda, into this substance which reacts, on the addition of caustic alkalies, with a red color, may take place, under certain circumstances, in a very short time. The elimination of the substance with the urine lasts, under certain conditions, a very long time. 6. Under the influence of santonin and santonin-soda the urine assumes readily a peculiar, yellow color. This color is evidently owing to the substance which is formed, in the animal body, out of the santonin. 7. On evaporating urine containing this substance, in a water bath, the latter is changed so much that it does not react any more with a red color on the addition of caustic potassa. 8. The urine secreted under the influence of santonin is not always of a saffron tint, but may occasionally assume a red color, viz., if ammonia is formed by decomposition of urea, or if the santonin has been administered in combination with alkalies. 9. Santonin and santonin-soda exert a remarkable influence on the brain and the organs of vision; they produce incoherency of ideas and chromatopsy. 10. The chromatopsy caused by santonin or santonin-soda is undoubtedly connected with the formation of the substance, reacting with a red color on addition of caustic potassa; we, therefore, call this condition "xanthopsy." The more the blood contains of this substance, the greater is the chromatopsy. 11. Chromatopsy can not be caused by dropping an aqueous solution of santonin-soda directly into the eyes. 12. The phenomena produced by santonin poisoning are different according to the difference of circumstances and conditions. Death is almost always preceded by convulsions.—(*Deutsche Klinik*, 1860, Nos. 27 and 28.)

2.—Messrs. v. Hasselt and Rienderhoff publish, in the *Nederl. Tijdsch. voor Geneesk.*, 1860, "a contribution to the toxicodynamic knowledge of santonin." They draw from their experiments the following conclusions, important to the practitioner as well as to the toxicologist: 1. Santonin can act as a poison. 2. As such it seems to belong, in general, to the class of *narcotica spinalia*, without leaving in the dead body any perceptible sign of its irritating secondary effect, although symptoms of it are observed in man during life. 3. Its action, on the administration of large doses, is analogous to that of tetanic poisons. 4. In relatively smaller doses, for instance, of six grammes, it produces in dogs slight symptoms of poisoning. 5. In large doses of sixty to ninety grammes, it can act fatally on these animals with relative rapidity. 6. Santonin manifests its action then, at first, in the sphere of the motory nerves, which action is shown by spasmodic contractions of the muscles without an increase of sensibility. The course of the affection makes it evident that the motory part of the spinal marrow is acted upon progressively from below upward. The fatal termination seems to be owing to spasms of the respiratory muscles and of the muscles of the larynx. These spasms may be considered the cause of the asphyxia which finally takes place. 7. The post-mortem appearances, hyperæmia of the lungs, engorgement of the heart, hyperæmia of the cerebro-spinal membranes, and capillary injection of the medulla spinalis and oblongata, are probably in causal relation to the spasmodic contractions of the muscles as well as to the death by asphyxia.

Without having the intention of assuming, from their experiments, that the effects upon man are equal to those upon dogs, the authors still believe that, considering their observations in regard to the action of santonin upon man, they have sufficient reasons to conclude *that santonin is, by no means, to be regarded as an innocent remedy.*—(*Medizinische Neuigkeiten*, August 18th, 1860.)

---

### Medical Chemistry.

I.—*On the Products of Decomposition of Albuminoid Substances.* By MM. Erlenmeyer and Schoeffer.

The authors have endeavored to determine, *quantitatively*, the products of the decomposition of albuminoid substances, by sulphuric acid. The acid employed was diluted with one part and a half of water. The following are the first results of these researches :—

The best proportion of acid to be employed is that of five parts for an animal substance, with the exception of horn, which requires ten parts of acid. Three hours of boiling suffice to effect decomposition.

The ligaments of the nape of the neck have given one-quarter per cent. of tyrosin, and thirty-six, forty-one, and forty-five per cent. of leucin; a little of the latter remains in the mother liquids.

From fibrin of the blood, pure tyrosin is easily obtained by neutralizing first the liquid, then evaporating it to 1·08, 1·10 of density; crystallization takes place on cooling.

The following are the results obtained with a certain number of these matters :—

| | Leucin. | Tyrosin. |
|---|---|---|
| Fibrin of blood . . . . | 14 per cent. | 2 per cent. |
| Fibrin of muscles . . . | 1 " | 18 " |
| White of egg . . . . . | 1 " | 10 " |
| Horn . . . . . . | 10 " | 3·6 " |

Besides leucin and tyrosin, the authors have found in fibrin, albumen, and casein the still badly-defined body which has been observed by Bopp among the products of decomposition of these proteinaceous substances, (*Annuaire de Chimie*, 1850, p. 560,) and which Messrs. Erlenmeyer and Schoeffer are tempted to consider a mixture of several compounds, of which one, at least, contains sulphur.—(*Journ. für praktische Chem. and Journ de Pharmacie et de Chimie*, October, 1860.)

---

### Toxicology and Legal Medicine.

I. *On the Effects of Chronic Lead Poisoning on the Fœtus.* By M. Paul.

M. Paul's attention was first called to this subject by the case of a female type-polisher, who, prior to her exposure to habitual contact with lead, had borne three fine children; while of the ten pregnancies which had since then occurred, eight were attended with miscarriage, the only child born at full time dying when five months old. She informed him that almost all the women similarly employed either miscarried or were unable to rear their children. Investigating the subject further, he found that these statements were founded on fact; and that not only was this hereditary influence imparted by the mother who had been working with lead, but also by the father, when the mother herself had not been exposed to such influence. Of this latter fact he has not been able to obtain more than 7 instances; but altogether his paper is founded upon 81 cases, which he has investigated. In some instances the effect upon the fœtus has been produced after but slight exposure, and in which the death of the infant has been almost the sole accident resulting from such exposure. Of the 81 individuals, male and female, interrogated, in 29 pregnancies occurred while engaged in manipulating the lead, these pregnancies amounting in all to 123. The results were, 64 abortions, 4 premature labors, and 5 born dead; that is, 73 children dead prior to delivery! Moreover, 20 children died in the course of the first year, 8 in that of the second, 7 in that of the third, and 1 later. There are 14 living children, but 10 only more than three years of age. In 15 instances, too, the women suffered from metrorrhagia, which was probably, in fact, abortion—for the saturine poisoning, in the degree in which it causes the death of the product of conception, does not usually disturb the menstruation itself, either in girls, in persons who have not borne children, or in the intervals of pregnancies. The author is still engaged in investigating the entire subject, but in the mean time desires to call attention to what he believes to be a new subject of inquiry.—(*Med. Times*, August 18, 1860, from *Archives Générales*, May, 1860.)

II. *Sudden Death without any adequate Post-mortem Appearances.* By Mr. J. Z. Laurence.

Mr. J. Z. Laurence communicated to the Harveian Society, May 3, 1860, the following case and interesting remarks:—

"On the twenty-fourth of April, the housemaid in a medical man's family was sent out to fetch the beer, when, within ten minutes afterward, she was found by the cook lying apparently dead in the scullery. I saw her myself in about another quarter of an hour. There was no action of the heart, no breathing, no sensibility; the body was still warm; the face livid and swollen. The only symptoms observed during life were that, for about a week before her death, she suffered from earache, appeared unusually indolent and stupid, and at times complained of faintness.

"*Post-mortem examination*, about twenty-two hours after death. The body was large, finely developed, very fat. Cadaveric rigidity was strongly marked in the limbs and the firmly-clenched lower jaw. The left ventricle of the heart was filled with dark fluid blood, and was firmly contracted; the right was flaccid and collapsed, containing but a very small clot. The muscular substance was apparently normal. The heart was altogether small in proportion to the body. The lungs were slightly congested. Between the two openings of the stomach was an oblong patch of fine punctiform florid injection in the lines of the rugæ. It contained an undigested meal of some stewed steak, of which the patient had partaken shortly before death. No poison of any kind was found in a careful analysis instituted by Mr. Rodgers, at the request of the jury. The intestines were not opened; their peritoneal coat was covered with congested veins, but in no very marked degree. The liver was somewhat congested. The spleen was soft and congested. The kidneys were very much congested. (She was menstruating at the time of her death.) The superficial veins of the brain, as those of the medulla oblongata, were turgid with dark blood. There was a slight amount of serum beneath the arachnoid, (which was somewhat opaque here and there,) especially in the basilar fossa. The brain-substance itself was perfectly normal. The larynx, trachea, and œsophagus were healthy, and contained no traces of any food in them.

"*Remarks.*—This case is interesting in medical jurisprudence, in two points of view: first, for the unsual precision with which the entire suddenness of the death could be fixed; second, for the total absence of any *post-mortem* appearances usually found in cases of sudden death, (such as heart disease, burstiug of aneurisms, etc.) In the *Annales d'Hygiène Publique* for 1838 will be found a paper by Devergie, entitled 'De la Morte Subite,' in which, with other causes of sudden death, he signalizes that by syncope, which he had found to occur three times in forty cases. Dr. Tourdés reported to the scientific congress meeting held at Strasbourg in 1842, that he had met with this cause of sudden death once in twenty-six cases. It appears to be identical with the 'idiopathic asphyxia' of Chevalier. Devergie, in the paper above refered to, gives the following as the post-mortem appearances he found in his three cases of death from syncope: '1. The absence of all congestion of the organs. 2. The normal state of all the organs. 3. The existence of about an equal quantity of blood in the right and left cavities of the heart, regard being had to their respective sizes. 4. Perhaps the coagulation of the blood in the fibrinous state.' As another cause of sudden death, Devergie further indicates that arising from cerebral congestion, which may, he says, be limited to the meninges. In illustration of this, he relates the case of a man aged sixty-three, who, having only complained of slight headache in the morning, took a good breakfast, after which he slept for two hours, and, on awaking, suddenly died. No congestion of the brain-substance was found; there was an old apoplectic clot in the left corpus striatum; there was great congestion of the meninges of the brain and spinal cord.

"Directing attention now to the condition of the stomach in our case, we may naturally ask ourselves whether the undigested meal, or the patch of injection on its coats, may have had anything to do with the cause of death? As regards the latter, no one would, I think, after the researches of Dr. Yellowly and others, attach any great independent value to mere vascularity of the gastric mucous membrane. The undigested meal may perhaps possess rather more significance. The deceased seems to have been in the habit of 'bolting' her meals very rapidly. The stewed steak found in her stomach, I am informed, she partook of stealthily—and therefore probably hastily—before her proper dinner, shortly before death.

"Dr. Taylor, at page 152 of the second edition of his work on *Poisons*, has cited several instances of persons dying quite suddenly, with nothing to account for the death but distention of the stomach; but I noticed no such distention in the present case. We may not unreasonably connect these conditions of the stomach with the syncopal affection. Professor Weber has shown that irritation of the pneumogastrics will stop the heart's action; and my distinguished friend and colleague, Dr. Brown-Séquard, has further shown that crushing the semilunar ganglia has a similar effect on the heart, provided the great splanchnic and pneumogastric nerves remain intact.

"I should feel disposed to regard the present case as one of mixed nature, presenting some of the signs of syncopal asphyxia, others of apoplexy. Explain it, however, as we may, it still remains one of considerable interest, proving as it does that, under certain (as yet very imperfectly understood) circumstances, death may occur suddenly without leaving any traces at all adequate (in our present state of knowledge) to account for the fatal result."—(*British Medical Journal*, May 19, 1860.)

III. *Remarks on Fish Poisons.* By DR. REIL.

The observations made by the author on this subject are, essentially, the following:—

Fish may prove injurious to health either in the preserved condition, that is, salted, pickled, or dried, or in the fresh state. Their poisonous properties in the simple or fresh state may be owing to the following circumstances: 1. They may themselves under all circumstances be poisonous, as has been maintained in regard to several species of fishes, although the fact requires further proofs. 2. They may under certain circumstances acquire poisonous properties, as for instance, by diseases peculiar to them, or at certain seasons of the year, or, as is known of the *Cyprius barba* and *Cyprius carpio*, at the spawning season. 3. Poisonous substances, such as *Hydrocarpus inebrians*, *Menispernum cocculum*, *Delphinium staphysagria*, etc., may have been used in catching them, or they may have been poisoned in another manner—for instance, by acids and metallic salts which contaminate the water in the neighborhood of factories. 4. Their injurious properties may be owing to commencing putrefaction.

The different modes of preserving fish give rise to chemical processes which cause the formation of poisonous matters. Injurious consequences from eating salted fish, particularly salted sturgeon, are frequently observed in Russia. But also codfish, and the smaller kinds of preserved fish, have given rise to symptoms of poisoning. Cases of poisoning from herrings are perhaps the rarest, and this is probably owing to the fact that the time for catching herrings is limited to a certain season, that they are salted without delay when still at sea, and that they are more rapidly consumed.

The character of the poison generated in preserved fish is still doubtful; at first the poisonous substance was thought to be of cryptogamous growth, or a fatty acid; more recently Schlossberger advanced the view that the propylamin (trimethylamin) contained in the brine is the poison in question, but this opinion has been refuted by the experiments of Buchheim.—(*Casper's Vierteljahrschrift*, xv. 2, 1860.)

